# Predicting Pathological Complete Response After Neoadjuvant Chemotherapy in Advanced Breast Cancer by Ultrasound and Clinicopathological Features Using a Nomogram

**DOI:** 10.3389/fonc.2021.718531

**Published:** 2021-11-23

**Authors:** Hao Cui, Dantong Zhao, Peng Han, Xudong Zhang, Wei Fan, Xiaoxuan Zuo, Panting Wang, Nana Hu, Hanqing Kong, Fuhui Peng, Ying Wang, Jiawei Tian, Lei Zhang

**Affiliations:** ^1^ Department of Ultrasound Medicine, The Second Affiliated Hospital of Harbin Medical University, Harbin, China; ^2^ Department of General Surgery, The Second Affiliated Hospital of Hebei Medical University, Shijiazhuang, China

**Keywords:** breast cancer, neoadjuvant chemotherapy, ultrasound, pathological complete response, nomogram

## Abstract

**Background and Aims:**

Prediction of pathological complete response (pCR) after neoadjuvant chemotherapy (NAC) for breast cancer is critical for surgical planning and evaluation of NAC efficacy. The purpose of this project was to assess the efficiency of a novel nomogram based on ultrasound and clinicopathological features for predicting pCR after NAC.

**Methods:**

This retrospective study included 282 patients with advanced breast cancer treated with NAC from two centers. Patients received breast ultrasound before NAC and after two cycles of NAC; and the ultrasound, clinicopathological features and feature changes after two cycles of NAC were recorded. A multivariate logistic regression model was combined with bootstrapping screened for informative features associated with pCR. Then, we constructed two nomograms: an initial-baseline nomogram and a two-cycle response nomogram. Sensitivity, specificity, negative predictive value (NPV), and positive predictive value (PPV) were analyzed. The C-index was used to evaluate predictive accuracy.

**Results:**

Sixty (60/282, 21.28%) patients achieved pCR. Triple-negative breast cancer (TNBC) and HER2-amplified types were more likely to obtain pCR. Size shrinkage, posterior acoustic pattern, and elasticity score were identified as independent factors by multivariate logistic regression. In the validation cohort, the two-cycle response nomogram showed better discrimination than the initial-baseline nomogram, with the C-index reaching 0.79. The sensitivity, specificity, and NPV of the two-cycle response nomogram were 0.77, 0.77, and 0.92, respectively.

**Conclusion:**

The two-cycle response nomogram exhibited satisfactory efficiency, which means that the nomogram was a reliable method to predict pCR after NAC. Size shrinkage after two cycles of NAC was an important in dependent factor in predicting pCR.

## Introduction

With approximately 279,000 new cases diagnosed each year in China, breast cancer is a serious concern among the female population (1). Neoadjuvant chemotherapy (NAC) prior to surgery has already become the standard treatment in advanced breast cancer (2). NAC has proven to have many advantages (3). It can improve the quality of surgery for patients who were initially unable to undergo surgery, and it increases the likelihood of breast conservation surgery (4). Results from the National Surgical Adjuvant Breast and Bowel Project B-18 trial and European Organization for Research and Treatment of Cancer Trial 10902 established the equivalency of NAC and adjuvant chemotherapy regarding disease-free and overall survival and demonstrated that NAC has the added advantage of increasing breast conservation rates with acceptable local control (5); what is more, pathological complete response (pCR) after NAC is considered to be a better outcome (6). Breast cancer is a heterogeneous disease, and approximately 20% of breast cancers are resistant to NAC; hence, early response prediction of pCR might enable the selection and alteration of treatment strategies (7, 8).

To date, the main evaluation methods for NAC are the Miller and Payne System (MP) (9) and Response Evaluation Criteria in Solid Tumors (RECIST) (10). RECIST evaluates the curative effect of NAC mainly according to changes in tumor size (10). The MP system is the gold standard for NAC, yet results can be obtained only after the entire course of NAC (9). Therefore, an early, non-invasive, and comprehensive information evaluation system to predict whether pCR is likely to occur is urgently needed. NAC-related studies have used various imaging modalities to assess tumor response in breast cancer, including MRI, [^18^F]FDG PET, mammography, and ultrasound. Hatt et al. suggested that the reduction of primary tumor measurements after two cycles of NAC of the metabolically active volume of [^18^F]FDG PET predicts histopathologic tumor response with higher accuracy (11). Kim investigated that using stringent MRI criteria could accurately predict pCR in advanced breast cancer after NAC (12). Anderson showed that optical mammography is a reliable method to predict drug response to NAC at the therapy midpoint to guide further decision making for NAC (13).

Among these radiology methods, breast ultrasound has many advantages, including safety, low cost, and wide use (14). It is worth noting that ultrasound plays a great role in evaluating the tumor response to NAC. Keune et al. observed that ultrasound was more accurate than mammography in predicting residual tumor size following NAC (15). Savaridas considered that there was a small size in ultrasound and mammographic spiculation (16). Some studies have reported that elasticity ultrasound may be used to assess the response to NAC in advanced breast cancer as early as 2 weeks into treatment, with high sensitivity and specificity (17). However, there is no currently available tool that can integrate multiple ultrasounds into a single numerical estimate of tumor response in NAC.

Nomograms can integrate several important factors to predict a specific endpoint in a graphical representation (18) and have been shown to be more accurate than conventional staging systems (19). In this context, we aimed to develop and validate a nomogram (the baseline-based nomogram) based on multi-ultrasound features and clinicopathological features to predict pCR after NAC. Based on the tumor response of effective cases after two cycles, which may be the most obvious (20), we also developed another nomogram (two-cycle response nomogram) incorporating the changes in the ultrasound features to indicate the initial response to NAC.

## Materials and Methods

### Patients

**Figure 1:**
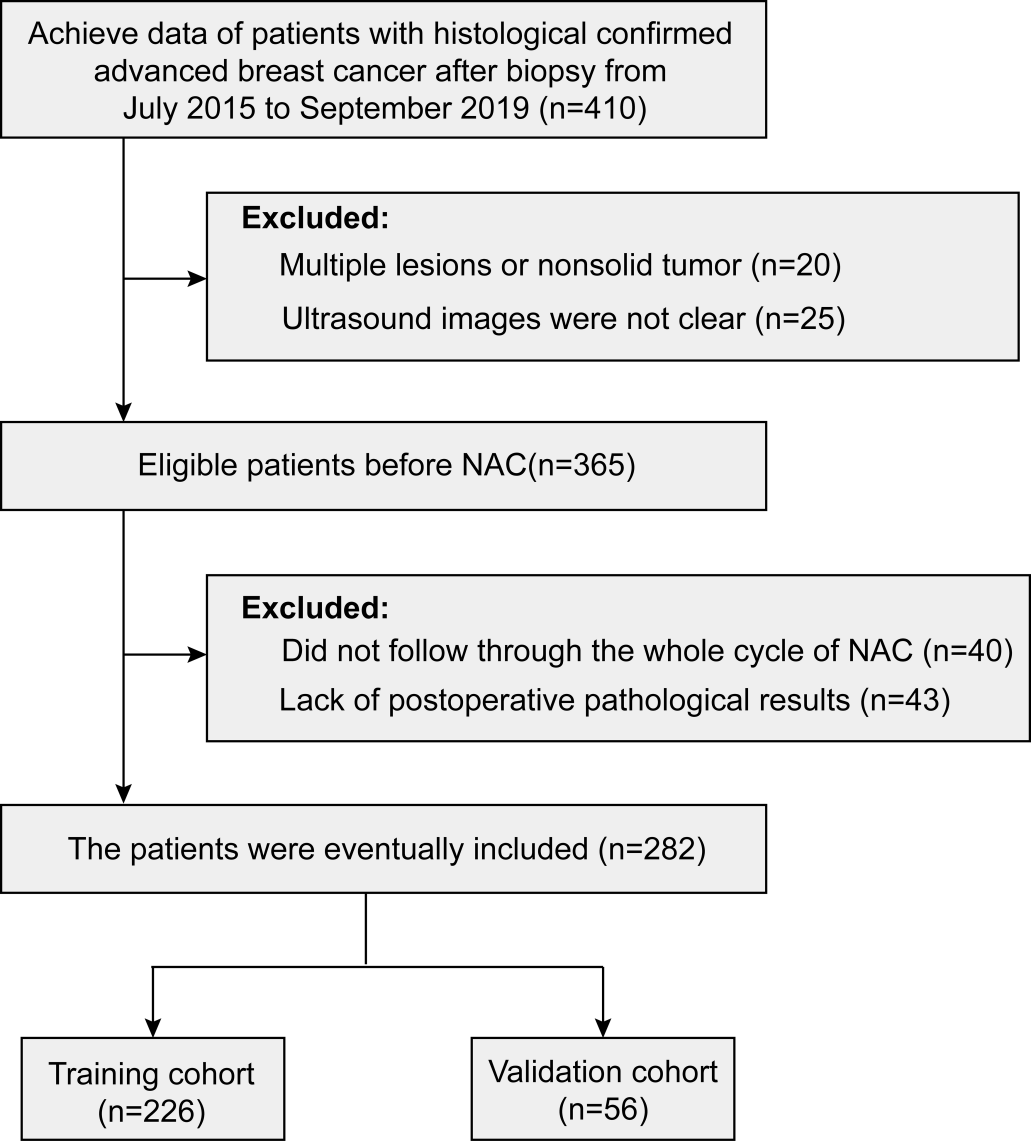
Recruitment pathway for patient selection.

This study was approved by the institutional ethics committee. From July 2015 to September 2019, patients with breast lesions initially underwent ultrasound examination and then underwent ultrasound-guided biopsy for pathological examination. According to the guideline, patients with advanced breast cancer suitable for NAC were selected. It is worth mentioning that patients who received complete cycles of NAC were enrolled (n = 282). The process for selecting patients for model development is presented in **Figure 1**.

According to the guidelines and standards for the diagnosis and treatment of breast cancer by the Chinese Anti-Cancer Association, 64 patients received doxorubicin (60 mg/m^2^ i.v.) and docetaxel (100 mg/m^2^ i.v.) in six cycles; 50 patients received doxorubicin (60 mg/m^2^ i.v.) and cyclophosphamide (500 mg/m^2^ i.v.) for four cycles; 142 patients with HER2-positive tumors received doxorubicin (60 mg/m^2^ i.v.), cyclophosphamide (250 mg/m^2^ i.v.), docetaxel (100 mg/m^2^ i.v.), and trastuzumab (8 mg/kg) in four cycles; and 26 patients were treated with doxorubicin (60 mg/m^2^ i.v.) and cyclophosphamide (500 mg/m^2^ i.v.) in four cycles, followed by docetaxel in four cycles.

Before NAC, we collected clinical data (age and size), ultrasound data (tumor shape, orientation, boundary, halo, posterior acoustic, calcification, margin, echo pattern, vascularity, elasticity score, and Breast Imaging-Reporting and Data System (BI-RADS)), and pathological data (estrogen receptor (ER), progesterone receptor (PR), HER2 status, Ki67 status, and pathology stage). After the two cycles, we recorded the change in size and any changes in all ultrasound features. Ultimately, 282 patients were enrolled in this study.

### Ultrasound Examination

We performed ultrasound scans before NAC and after two cycles of NAC and then recorded the initial size and ultrasound features of the lesions. In addition, we recorded the changes in size and ultrasound features after two cycles of NAC. This study involved multi-ultrasound, including B-mode ultrasound, Doppler ultrasound, and elastic ultrasound. These ultrasound images were obtained with a HITACHI Vision 900 system (Hitachi Medical System, Tokyo, Japan) equipped with a linear probe of 6–13 MHz. All real-time scanning was performed by two radiologists with 5 years of experience in breast ultrasonography. We scanned the whole mass with B-mode ultrasound to find and record the section of the maximum diameter. The static images and cine clips of multi-ultrasound were saved in the database for specific ultrasound feature analysis. Three breast radiologists with more than 10 years of clinical experience reviewed the ultrasound images retrospectively and independently. A consensus interpretation was reached in cases of disagreement. The grayscale ultrasound criteria were assessed by using the standardized lexicon for ultrasound BI-RADS (21). Blood flow was evaluated using the Adler grade classification (22). The elastic ultrasound criteria were according to the World Federation for Ultrasound in Medicine and Biology (WFUMB) guidelines (23).

### Histological Examination

Before NAC, the tissues were obtained by needle biopsy for the pathology. The tissues were formalin-fixed, paraffin-embedded, and subsequently used for immunohistochemistry (IHC) staining with the appropriate antibodies. According to the guideline of 2019 WHO classification (24), breast tumors were classified into corresponding histological types and grade. The cutoff points for ER-positive, PR-positive, and P53 were 1%. HER2 status was graded as 0, 1+, 2+, and 3+. Only 3+ was deemed to be positive, whereas 0 and 1+ were deemed to be negative. Samples with a <2-fold change in fluorescence *in situ* hybridization (FISH) were regarded as negative, and those with >2-fold increase were regarded as positive. Ki67 was visually scored for the percentage of tumor cell nuclei with positive immunostaining above background. Over 14% was considered high expression, and less than 14% was considered low expression (25).

### Definition of Pathological Complete Response

Pretreatment core biopsies and surgical specimens after surgery were used to evaluate the response to chemotherapy. The pathological diagnosis and response to NAC were determined by a breast pathologist with 10 years of experience in breast pathology using the MP system of five grades based on malignant cell changes between the biopsy tissue and surgical specimen (9). pCR was classified as the absence of any invasive cancer cells in the tumor bed at surgical resection after whole cycles of NAC and absence of nodal metastases at axillary surgery.

### Molecular Subtypes of Breast Cancer

The breast cancer molecular subtypes were categorized according to the IHC results for ER, PR, HER2, and Ki-67 status, as recommended by the 12th International Breast Conference (26):

Luminal A (LA): ER or/and PR positive, HER2 negative, and Ki-67 <14%

Luminal B (LB): ER or/and PR positive, HER2 negative, and Ki-67 ≥14%, ER or/and PR positive and HER2 overexpressed or/and amplified

HER2 amplified (HER2): ER and PR negative and HER2 overexpressed or/and amplified

Triple-Negative (TN): ER, PR, and HER2 negative

### Data Analysis and Statistical Analysis

#### Development of Nomogram Model

We randomly extracted 226 patients (80% of the total cohort) from 282 as the training set; the remaining 20% served as the external validation cohort. Then, we used bootstrap sampling (1,000 times, random extract 80% cases every time) to train the models based on the training cohort. Multivariate Cox regression analysis was used to screen the significant characteristics related to pCR in initial-baseline patients and two-cycle response patients. Based on the 1,000 times bootstrap sampling, the 1,000 cohorts with significant characteristics were obtained from the multivariate Cox regression analysis. The frequency of each characteristic was calculated, and characteristics greater than 500 times were regarded as informative characteristics. Finally, we constructed two nomograms (initial-baseline and two-cycle responses) based on these informative characteristics.

#### Nomogram Validation

To evaluate the predictive effect of the nomograms, the model needed to be verified. The internal evaluation of the nomogram was performed with the bootstrap self-sampling method, and the external validation was performed with the validation cohort. In the verification process, the C-index was used to evaluate the performance of the nomogram using the validation cohort, and a calibration curve was drawn to evaluate its calibration effect.

#### Baseline Statistical Analysis

Pearson’s chi-square test and independent t-tests were used for comparisons between the training cohort and validation cohort. Multivariate Cox regression analysis was used to screen the significant characteristics.

**Figure 2:**
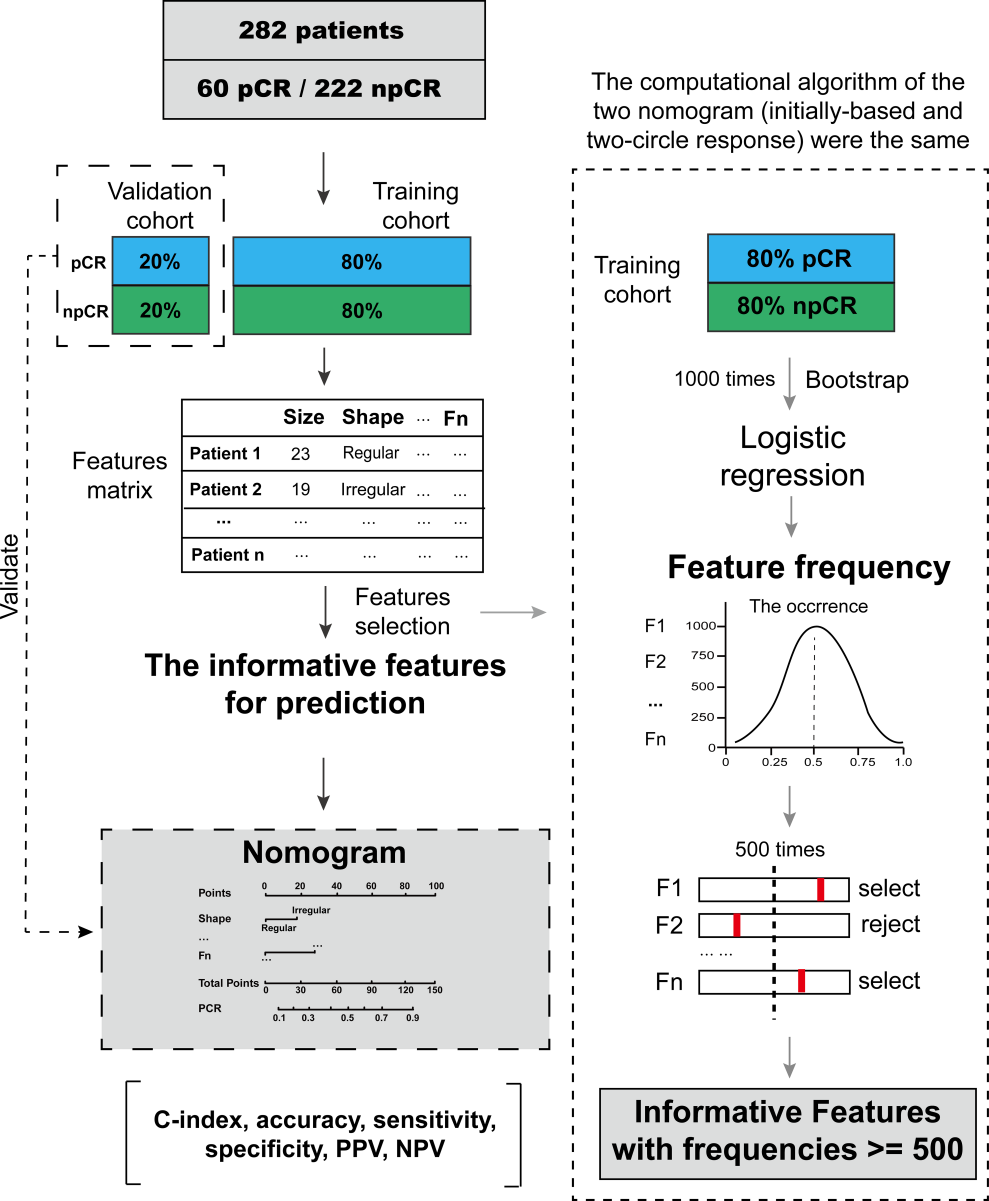
Step-by-step protocol for the nomogram computational algorithm.

All the statistical analyses were performed with R software (R Foundation for Statistical Computing, Vienna, Austria; http://www.R-project.org, 2016). The following R packages were used: the “survival” package was used for multivariate logistic regression. The “rms” package was used for nomograms, calibration curves, and C-index. All statistical tests were two-sided, and *p* < 0.05 was considered significant. The study flowchart is shown in **Figure 2**.

## Results

### Patient Characteristics

In our study, 60 of the 282 patients have achieved pCR, and the proportion was 21.28% (60/282). The ultrasound and clinicopathological characteristics of the initial-baseline and two-cycle NAC are summarized in **Table 1**. The cohorts of the training cohort and validation cohort are summarized in **Tables S1**, **S2**, respectively. There were no differences in the ultrasound and clinicopathological characteristics between the training and validation cohorts in either initial-baseline patients or two-cycle response patients. The rate of obtained pCR was 20.8% (47/226) in the training cohort and 23.1% (13/56) in the validation cohort, and there were no significant differences between the two cohorts (**Table S3**, *p* = 0.70).

**Table 1:** Ultrasound and clinicopathological characteristics of patients of initial-baseline patients and two-cycle response patients.

Characteristics	Initial-baseline patients (n = 282)		Two-cycle response patients (n = 282)		
	n	Percent (%)	Characteristics changes	n	Percent (%)
**Age (years)**					
Mean ± SD	51.8 ± 9.4				
**Subtype**					
TNBC	64	22.70			
HER2	69	24.47			
Luminal A	76	26.95			
Luminal B	73	25.89			
**Pathological grade**					
I	8	2.84			
II	192	68.09			
III	82	29.08			
**Ki-67 (%)**					
Mean ± SD	20.7 ± 17.7				
**Tumor size (mm)**			Size (mm)		
Mean ± SD	32.2 ± 7.9		Increase	0	
			Shrinkage	4.7 ± 4.7	
**Shape**			**Shape**		
Regular	51	18.09	No change	253	89.72
Irregular	231	81.91	Change	29	10.28
**Orientation**			**Orientation**		
Parallel	88	31.21	No change	252	89.36
Vertical	194	68.79	Change	30	10.64
**Boundary**			Boundary		
Circumscribed	28	9.93	No change	242	85.82
Indistinct	254	90.07	Change	40	14.18
**Echogenic halo**			**Echogenic halo**		
Absent	182	64.54	No change	254	90.07
Present	100	35.46	Disappear	28	9.93
			Appear	0	0
**Margin**			**Margin**		
Smooth	64	22.70	No change	251	89.01
Lobulated	87	30.85	Change	31	10.99
Angular	131	46.45			
**Posterior acoustic**			**Posterior acoustic**		
Enhancement	124	43.97	No change	189	67.02
No change	102	36.17	Change to shadowing	93	32.98
Shadowing	56	19.86	Change to enhancement	0	0
**Calcification**			**Calcification**		
Absent	141	50.0	No change	258	91.49
Present	141	50.0	Disappear	24	8.51
			Appear	0	0
**Echo pattern**			**Echo pattern**		
Hypoechoic	277	98.23	No change	252	89.36
Mixed-echoic	3	1.77	Change	30	10.64
**Adler degree**			**Adler degree**		
0	32	11.35	No change	252	89.36
1	79	28.01	Reduce	30	10.64
2	66	23.40	Increase	0	0
3	105	37.23			
**BI-RADS**			**BI-RADS**		
1	1	0.35	No change	249	88.30
3	1	0.35	Reduce	33	11.70
4	37	13.12	Increase	0	0
5	243	86.17			
**Elasticity score**			**Elasticity score**		
4	100	35.46	No change	205	72.70
5	182	64.54	Reduce	77	27.30
			Increase	0	0

TNBC, triple-negative breast cancer; BI-RADS, Breast Imaging-Reporting and Data System.

### Identifying Independent Predictors of Pathological Complete Response Using Multivariate Logistic Regression

Among the entire cohort, triple-negative breast cancer (TNBC) and HER2-amplified patients were more likely to obtain pCR than luminal A and luminal B patients (*p* = 0.046). Among the 60 patients who achieved pCR, 19 were TNBC patients (31.7%, 19/60), 18 were HER2-amplified patients (30.0%, 18/60), 12 were luminal A patients (20.0%, 12/60), and 11 were luminal B patients (18.3%, 11/60).

In the training cohort of initial-baseline patients, the subtype, posterior acoustic pattern, and elasticity score were identified as independent predictors of pCR by multivariate logistic regression (**Table 2**). Among 47 patients who achieved pCR in the training cohort, 27 patients had enhancement (57.5%), 15 patients had no change (31.9%), and five patients had shadowing (10.6%) (*p* = 0.04). The elasticity score was commonly high (score was 5) in pCR patients (high degree *vs*. low degree, 64.2% *vs*. 35.8%, *p* = 0.02).

**Table 2:** Multivariate logistic analysis of pCR in initial-baseline and two-cycle response patients based on the training cohort.

Characteristics and the changes after two-cycle NAC	Initial-baseline patients		Two-cycle response patients	
	OR (95% CI)	*p*-Value	OR (95% CI)	*p*-Value
Subtype	0.68 (0.46, 0.99)	0.05*		
Ki-67	0.98 (0.96, 1.01)	0.13		
Pathological grade	1.37 (0.60, 3.10)	0.46		
Age	0.98 (0.94, 1.02)	0.27		
Tumor size	1.02 (0.97, 1.06)	0.45	1.20 (1.10, 1.31)	<0.001*
Shape	0.61 (0.18, 2.13)	0.44	1.50 (0.46, 4.86)	0.50
Orientation	1.29 (0.57, 2.91)	0.54	1.19 (0.32, 4.42)	0.78
Boundary	5.09 (0.7, 37.07)	0.11	1.31 (0.49, 3.54)	0.59
Echogenic halo	0.85 (0.40, 1.81)	0.67	2.4 (0.800, 7.17)	0.12
Margin	0.84 (0.48, 1.46)	0.54	1.77 (0.56, 5.54)	0.33
Posterior acoustic pattern	0.60 (0.36, 0.98)	0.04*	2.16 (1.02, 4.61)	0.05*
Calcification	0.70 (0.34, 1.43)	0.33	1.17 (0.33, 4.16)	0.81
Echo pattern	0.00 (0, Inf)	0.99	0.86 (0.26, 2.90)	0.81
Adler degree	1.11 (0.77, 1.60)	0.59	1.52 (0.51, 4.54)	0.45
BI-RADS	1.39 (0.47, 4.07)	0.55	1.71 (0.54, 5.47)	0.36
Elasticity score	2.61 (1.15, 5.94)	0.02*	2.48 (1.13, 5.41)	0.02*

pCR, pathological complete response; NAC, neoadjuvant chemotherapy; BI-RADS, Breast Imaging-Reporting and Data System.

^*^p < 0.05.

In the training cohort of two-cycle response patients, shrinkage, posterior acoustic changes to shadowing, and reduced elasticity scores were identified as independent predictors of pCR by a multivariate logistic regression model (**Table 2**). Size shrinkage was a particularly good independent factor for predicting pCR. As shown in **Figure 2**, shrinkage accounted for the largest effect proportionally: the greater the tumor size shrinkage, the more prone the patient was to achieve pCR (*p* < 0.001). Similar to size shrinkage, the reduction in elasticity score was associated with an increased likelihood of pCR (*p* = 0.016). Posterior acoustic changes to shadowing were also associated with pCR (*p* = 0.05).

### Nomogram Construction Using the Training Cohort

**Figure 3:**
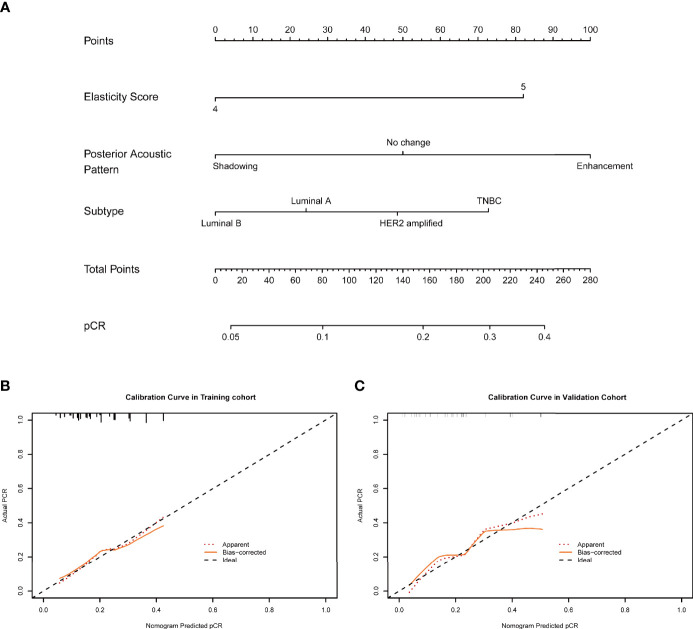
**(A)** An initial-baseline nomogram was constructed from three informative features (**Table 2**). To calculate the probability of patients achieving pCR after NAC, points for each parameter were assigned by corresponding values from the “points” axis, and the sum of the points was plotted on the “total points” axis. The probability of achieving pCR after NAC was the value at a vertical line from the corresponding total points **(B, C)**. pCR, pathological complete response; NAC, neoadjuvant chemotherapy.

**Figure 4:**
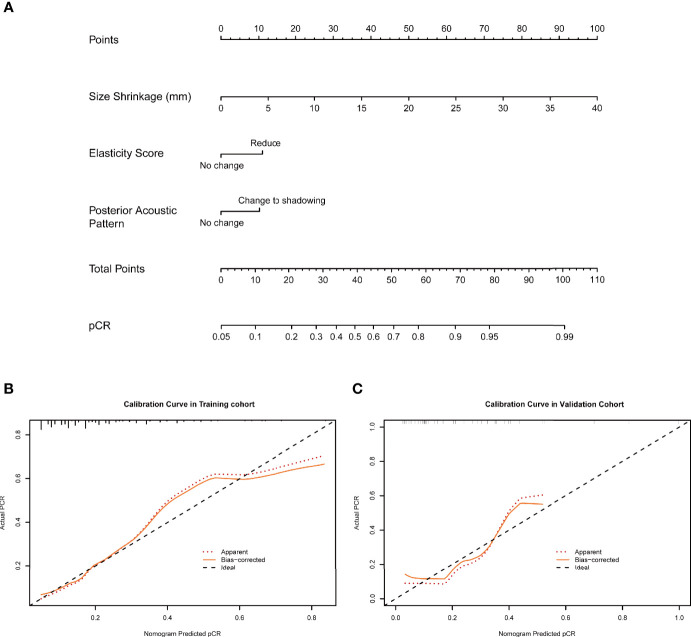
**(A)** A two-cycle response nomogram including early response to chemotherapy was constructed from three informative features (**Table 2**). When the total points reach 100, the probability of achieving pCR reached 99%, which mean that the prediction efficiency was satisfactory **(B, C)**. pCR, pathological complete response.

**Figure 5:**
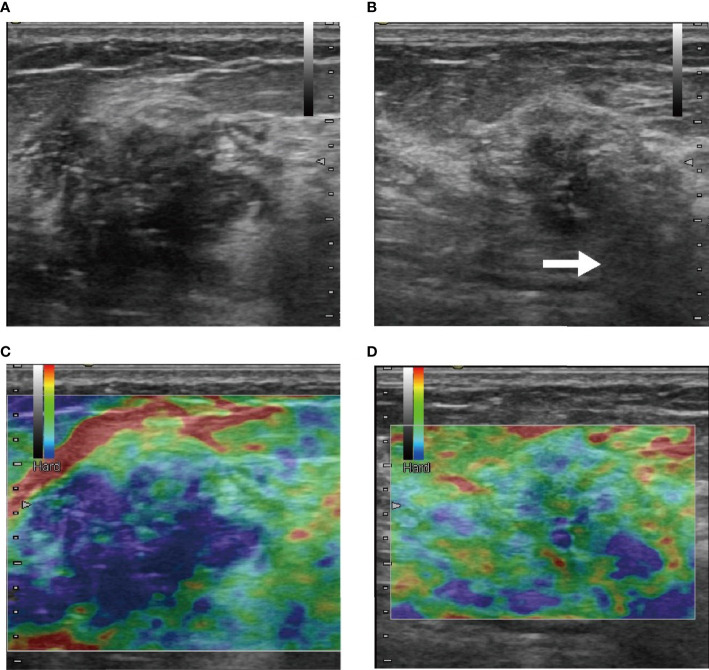
These were B-mode **(A, B)** and elasticity ultrasound **(C, D)** images of a patient who was treated with NAC and who eventually achieved pCR. **(A, C)** The initial images before NAC. **(B, D)** Images after two-cycle response. In B-mode images **(A, B)**, size shrinkage was obvious, and the posterior acoustic changed to shadowing (white arrow) after the two-cycle response was visible. From the elasticity ultrasound images **(C, D)**, we observed that the range of rendering blue was reduced, which mean that the elasticity score was reduced.

The nomogram was constructed based on significant factors from the multivariate logistic regression analysis using the training cohort to predict pCR (**Figures 3A** and **4A**). In the nomogram, the estimated probability of pCR could be achieved by summing the scores of each variable and locating them on the total score scale. For instance, one patient with a two-cycle response had a size shrinkage of 40 mm (100 points), elasticity score reduction (12 points), and posterior acoustic change to shadowing (12 points) of 124 points, which could be converted to a probability of pCR greater than 99.0%, which indicated a perfect model (**Figures 4A** and **5**).

### Validation and Performance of the Nomogram

In the training cohort, the C-index for the nomogram of the initial-baseline nomogram was 0.68 (95% CI: 0.59–0.76), while the two-cycle response nomogram was 0.78 (95% CI: 0.70–0.86). In the validation cohort, the C-index for the nomogram of the initial-baseline nomogram was 0.73 (95% CI: 0.59–0.87), while the two-cycle response nomogram was 0.79 (95% CI: 0.62–0.96).

**Figure 6:**
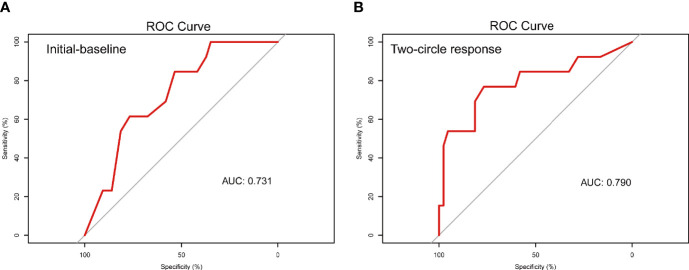
Receiver operating characteristic curve of the nomogram integrating ultrasound and clinicopathological features for predicting pCR after NAC. The AUCs of the initial-baseline nomogram **(A)** and two-cycle response nomogram **(B)** were 0.73 and 0.79, respectively. NAC, neoadjuvant chemotherapy; pCR, pathological complete response; AUC, area under the curve.

The two-cycle response nomogram showed the best discrimination in the validation cohort, with the area under the curve (AUC) reaching 0.79, and the AUC value was higher than that of the initial-baseline nomogram (AUC, 0.73) (**Figure 6**). These results suggested that the two-cycle response nomogram achieved favorable predictive efficacy. The calibration curves demonstrated good consistency in the training and validation cohorts (**Figures 3B, C** and **4B, C**). Therefore, our two-cycle response nomogram performed well.

In the validation cohort, the sensitivity and specificity of the two-cycle response nomogram were 0.77 (0.46–0.95) and 0.77 (0.61–0.88), respectively. The negative predictive value (NPV) of the initial-baseline nomogram and two-cycle response nomogram was perfect, with 0.85 (0.63–0.92) and 0.92 (0.74–0.96), respectively. The above results are shown in **Table 3**.

**Table 3:** The predictability of pCR by the nomogram of initial-baseline and two-cycle response.

	Initial-baseline nomogram	Two-cycle response nomogram
Sensitivity (95% CI)	0.62 (0.32–0.86)	0.77 (0.46–0.95)
Specificity (95% CI)	0.67 (0.52–0.81)	0.77 (0.61–0.88)
PPV (95% CI)	0.36 (0.23–0.69)	0.50 (0.33–0.85)
NPV (95% CI)	0.85 (0.63–0.92)	0.92 (0.74–0.96)

PPV, positive predictive value; NPV, negative predictive value; pCR, pathological complete response.

## Discussion

In the NAC setting, selecting patients likely to derive benefit from treatment is important and essential so that accurate assessment of early response to NAC can be used to tailor therapy (27, 28). Some randomized controlled trials demonstrated that those potential benefits could be used to assess response by ultrasound, and ultrasound has been commonly used for this purpose in clinical practice (29). However, few studies have developed multi-ultrasound models. In this study, we constructed two nomogram-based predictive models of ultrasound for patients with advanced breast cancer who were treated with NAC and validated each model. Notably, the two-cycle response nomogram performed favorably, with an AUC of 0.79 in the validation cohort. Therefore, the nomograms we conducted may be a useful tool in identifying patients who would respond well to NAC.

In this context, we constructed two nomograms to predict the pCR of NAC in advanced breast cancer. One was the initial-baseline nomogram, and the other was the two-cycle response nomogram. The initial-baseline nomogram was constructed using initial ultrasound features (elasticity score and posterior acoustic pattern) and clinicopathological features (subtypes); information on these features was readily available before treatment, while the lesion with large hardness (the elasticity score was 5) and enhancement posterior acoustic was prone to achieve pCR. From the nomogram, we could see that the maximum predictive value was 0.4, which means that the initial-baseline ultrasound characteristics could not predict pCR with sufficient certainty to recommend therapeutic modalities. It presented low sensitivity, specificity, and positive predictive value (PPV). These results could help us screen out cases that would not achieve pCR before treatment.

The most significant model obtained from this study was the two-cycle response nomogram. This nomogram was constructed using changes in ultrasound characteristics (size shrinkage, elasticity score, and posterior acoustic pattern), which means that the lesion with size shrinkage and hardness reduction (as the elasticity score reduced from 5 to 3) and the changing of posterior acoustic to shadowing was prone to achieve pCR. The C-index indicated good diagnostic efficiency with a value of 0.79. The sensitivity and specificity were satisfactory, with a value of 0.77 for both, and the NPV was extremely high, with a value of 0.92, implying that the two-cycle response nomogram performed favorably. These results could offer physicians and patients a general scope of prognosis at the time of two-cycle NAC.

Our data confirmed the increased likelihood of achieving pCR in HER2-amplified and TNBCs, and this result was similar to that of other studies (29). This may be because luminal breast cancers are slowly proliferating tumors that are more amenable to local treatment and because these patients also benefit from a much longer course of endocrine treatment (30). In contrast, HER2-amplified and TNBCs are rapidly proliferating tumors that are sensitive to NAC drugs (29).The size shrinkage after two-cycle NAC was the most important feature, taking the maximum weight in the two-cycle response nomogram constructed in our study. Previous studies revealed that early response to NAC after two cycles is a predictor of pCR and may therefore serve as a predictor for long-term outcome (31).

Our results indicated that informative ultrasound features, including posterior acoustic and elasticity scores, were associated with pCR. Posterior enhancement in the initial-baseline nomogram and posterior acoustic change to shadowing in the two-cycle response nomogram were both predicted to achieve pCR, especially the two-cycle response nomogram. Some research has revealed that tumors with posterior acoustic enhancement are more cellular and tend to rapidly proliferate because of the reduced attenuation of ultrasound waves compared with the surrounding tissue (32). In contrast, tumors with posterior acoustic shadowing might be formed by desmoplastic reactions that are more likely to occur in slowly proliferating tumors (33). These mechanisms indicated that rapidly proliferating tumors tend to achieve pCR, so the posterior acoustic system was enhanced initially and changed to shadowing after NAC. Elasticity ultrasound, as an imaging modality, would be practical and efficient in monitoring the response to NAC (17). Moreover, some scholars pointed out that the changes in stiffness of lesions after three cycles of NAC from baseline were strongly associated with pCR after six cycles (34). Those results were also reflected in this study. The high elasticity score before NAC and the reduction in elasticity score after two-cycle NAC were both associated with pCR. Tumor cells undergo “cellular retraction” during NAC, transforming from a single large mass to scattered small lesions (35) and reducing the stiffness of the lesion.

In total, we developed nomograms to predict pCR after NAC in located breast cancer. However, there existed some limitations in our study; firstly, the relatively small sample size of this study may have resulted in no molecular subtype performed. Future studies should divide patients by different molecular subtypes, especially the independent study targeted at hormone receptor-positive breast cancer with poor efficacy. It should also expand the scope of cases, such as non-advanced breast cancer, who need breast-conserving surgery. Secondly, the two-cycle response nomogram showed better performance in our study but still cannot predict the response to NAC before its administration, which would be the priority in our future study. Thirdly, we would add the results of breast exam *per se* efficiency to compare with the nomogram. What is more, we will carry out a prospective study to verify the effect of the nomogram in the future and, meanwhile, add more factors into the nomogram, such as lymphovascular invasion degree, the number of lymph node metastases, and the status of fibrosis.

## Conclusions

In conclusion, size shrinkage after two-cycle NAC was an important dependent factor to predict pCR. Ultrasound features and feature changes, including posterior acoustics and elasticity, were both associated with pCR. The two-cycle response nomogram exhibited satisfactory efficiency, which means that the nomogram was a reliable method to predict pCR after NAC. This should be further investigated, as it shows potential to serve as an imaging modality that would effectively predict pCR in real-world settings.

## Data Availability Statement

The original contributions presented in the study are included in the article/**Supplementary Material**. Further inquiries can be directed to the corresponding author.

## Ethics Statement

The studies involving human participants were reviewed and approved by the institutional ethics committee of Harbin Medical University (approval number, KY-2016-127). Written informed consent for participation was not required for this study in accordance with the national legislation and the institutional requirements.

## Author Contributions

HC and LZ designed this study. DZ, PH, and XuZ performed the search and collected the data. WF and XiZ checked the grammatical errors and typos. PW and FP detailed the statistical methods. YW, NH, and HK modified the introduction of the study. HK and LZ helped to revise the manuscript. All authors contributed to the article and approved the submitted version.

## Funding

This study was funded by the National Natural Science Foundation of China (grant number 82102072, 81701705, and 81974265), Postdoctoral Funding of Heilongjiang province (grant number LBH-Z17174) and Scientific research project of Heilongjiang Health Committee (grant number 2019-050), and the Fundamental Research Funds for the Provincial Universities (2020-KYYWF-1454).

## Conflict of Interest

The authors declare that the research was conducted in the absence of any commercial or financial relationships that could be construed as a potential conflict of interest.

## Publisher’s Note

All claims expressed in this article are solely those of the authors and do not necessarily represent those of their affiliated organizations, or those of the publisher, the editors and the reviewers. Any product that may be evaluated in this article, or claim that may be made by its manufacturer, is not guaranteed or endorsed by the publisher.
