# Supplementary material for: Predicting Pathological Complete Response After Neoadjuvant Chemotherapy in Advanced Breast Cancer by Ultrasound and Clinicopathological Features Using a Nomogram
Source: Front Oncol. 2021 Nov 23;11:718531. doi: 10.3389/fonc.2021.718531 (PMC8650158; doi:10.3389/fonc.2021.718531)
Supplement: Supplementary file 1 [file Table_1.docx]

**Supplemental Data**

Table S1 Characteristics of patients in training and validation cohorts of initial-baseline patients

| **Characteristics** | **Training cohort(n=226)** | **Validation cohort(n=56)** | **p value** |
| --- | --- | --- | --- |
| **Subtype** |  |  | 0.12 |
| TNBC | 46(20.35%) | 18(32.14%) |  |
| HER2 | 55(24.34%) | 14(25.00%) |  |
| Luminal A | 67(29.65%) | 9(16.07%) |  |
| Luminal B | 58(25.66%) | 15(26.79%) |  |
| **Ki-67** |  |  | 0.22 |
| Mean ± SD | 20.1±18.1 | 23.1±15.9 |  |
| **Pathological grade** |  |  | 0.16 |
| I | 7(3.10%) | 1(1.79%) |  |
| II | 159(70.35%) | 33(58.93%) |  |
| III | 60(26.55%) | 22(39.29%) |  |
| **Age** |  |  | 0.11 |
| Mean ± SD | 52.2±9.4 | 50.0±9.4 |  |
| **Tumor Size** |  |  | 0.68 |
| Mean ± SD | 32.1±7.7 | 32.6±8.7 |  |
| **Shape** |  |  | 0.60 |
| Regular | 39 (17.26%) | 12(21.43%) |  |
| Irregular | 187(82.74%) | 44(78.57%) |  |
| **Orientation** |  |  | 0.20 |
| Parallel | 75 (33.19%) | 13(23.21%) |  |
| Vertical | 151(66.81%) | 43(76.79%) |  |
| **Boundary** |  |  | 1.00 |
| Circumscribed | 22 (9.73%) | 6(10.71%) |  |
| Indistinct | 204(90.27%) | 50(89.29%) |  |
| **Echogenic halo** |  |  | 0.84 |
| Absent | 147(65.04%) | 35(62.50%) |  |
| Present | 79(34.96%) | 21(37.50%) |  |
| **Margin** |  |  | 0.10 |
| Smooth | 46(20.35%) | 18(32.14%) |  |
| Lobulate | 69(30.53%) | 18(32.14%) |  |
| angular | 111(49.12%) | 20(35.71%) |  |
| **Posterior acoustic** |  |  | 0.46 |
| Enhancement | 99(43.81%) | 25(44.64%) |  |
| No change | 79(34.96%) | 23(41.07%) |  |
| Shadowing | 48(21.24%) | 8(14.29%) |  |
| **Calcification** |  |  | 0.65 |
| Absent | 111(49.12%) | 30(53.57%) |  |
| Present | 115(50.88%) | 26(46.43%) |  |
| **Echo pattern** |  |  | 0.57 |
| Hypoechoic | 223(98.67%) | 54(96.43%) |  |
| Mixed-echoic | 3(1.33%) | 2(3.57%) |  |
| **Adler degree** |  |  | 0.18 |
| 1 | 29(12.83%) | 3(5.36%) |  |
| 2 | 58(25.66%) | 21(37.50%) |  |
| 3 | 55(24.34%) | 11(19.64%) |  |
| 4 | 84(37.17%) | 21(37.50%) |  |
| **BI-RADS** |  |  | 0.59 |
| 4 | 33(14.6%) | 6(10.71%) |  |
| 5 | 193(85.4%) | 50(89.29%) |  |
| **Elasticity score** |  |  | 0.91 |
| 4 | 81(35.84%) | 19(33.93%) |  |
| 5 | 145(64.16%) | 37(66.07%) |  |

Table S2 Characteristics of patients in training and validation cohorts of two-circle response patients

| **Characteristics changes** | **Training cohort(n=226)** | **Validation cohort(n=56)** | **p value** |
| --- | --- | --- | --- |
| **Size** |  |  | 0.19 |
| Mean ± SD | 4.80±4.80 | 4.00±4.30 |  |
| **Shape** |  |  | 0.39 |
| No change | 205(90.71%) | 48(85.71%) |  |
| Change | 21(9.29%) | 8(14.29%) |  |
| **Orientation** |  |  | 0.09 |
| No change | 206(91.15%) | 46(82.14%) |  |
| Change | 20(8.85%) | 10(17.86%) |  |
| **Boundary** |  |  | 1 |
| No change | 194(85.84%) | 48(85.71%) |  |
| Change | 32(14.16%) | 8(14.29%) |  |
| **Echogenic halo** |  |  | 1 |
| No change | 204(90.27%) | 50(89.29%) |  |
| Change | 22(9.73%) | 6(10.71%) |  |
| **Margin** |  |  | 0.52 |
| No change | 203(89.82%) | 48(85.71%) |  |
| Change | 23(10.18%) | 8(14.29%) |  |
| **Posterior acoustic pattern** |  |  | 0.76 |
| No change | 150(66.37%) | 39(69.64%) |  |
| Change of shadowing | 76(33.63%) | 17(30.36%) |  |
| Change of enhancement | 0 | 0 |  |
| **Calcification** |  |  | 0.23 |
| No change | 204(90.27%) | 54(96.43%) |  |
| Change | 22(9.73%) | 2(3.57%) |  |
| **Echo pattern** |  |  | 0.83 |
| No change | 201(88.94%) | 51(91.07%) |  |
| Change | 25(11.06%) | 5(8.93%) |  |
| **Adler degree** |  |  | 0.83 |
| No change | 201(88.94%) | 51(91.07%) |  |
| Change | 25(11.06%) | 5(8.93%) |  |
| **BI-RADS** |  |  | 0.37 |
| No change | 202(89.38%) | 47(83.93%) |  |
| Change | 24(10.62%) | 9(16.07%) |  |
| **Elasticity score** |  |  | 1.00 |
| No change | 164(72.57%) | 41(73.21%) |  |
| Reduce | 62(27.43%) | 15(26.79%) |  |
| Increase | 0 | 0 |  |

Table S3 The pCR rate of the training and validation cohorts

| **Status** | **Training** | **Validation** | **p value** |
| --- | --- | --- | --- |
| pCR |  |  | 0.70 |
| Non obtain | 179(79.2%) | 43(76.79%) |  |
| Obtain | 47(20.8%) | 13(23.21%) |  |
